# Fast Bulk Depolymerization of Polymethacrylates by ATRP

**DOI:** 10.1021/acsmacrolett.3c00389

**Published:** 2023-08-02

**Authors:** Ferdinando De Luca Bossa, Gorkem Yilmaz, Krzysztof Matyjaszewski

**Affiliations:** Department of Chemistry, Carnegie Mellon University, 4400 Fifth Avenue, Pittsburgh, Pennsylvania 15213, United States

## Abstract

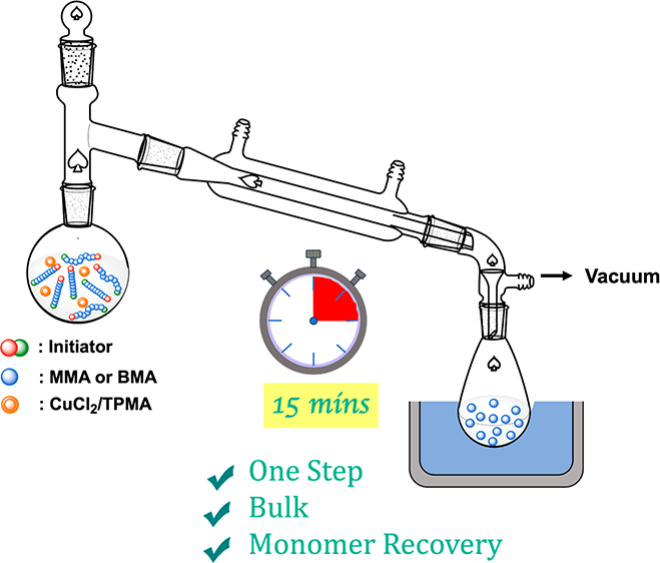

Fast bulk depolymerization of poly(*n*-butyl methacrylate) and poly(methyl methacrylate), prepared by atom transfer radical polymerization (ATRP), is reported in the temperature range between 150 and 230 °C. Depolymerization of Cl-terminated polymethacrylates was catalyzed by a CuCl_2_/TPMA complex (0.022 or 0.22 equiv vs P-Cl) and was studied using TGA, also under isothermal conditions. Relatively rapid 5–20 min depolymerization was observed at 230 and 180 °C. The preparative scale reactions were carried out using a short-path distillation setup with up to 84% depolymerization within 15 min at 230 °C.

Sustainable chemistry encompasses designing, manufacturing, and employing efficient, safe, and environmentally benign chemical products and processes. It also includes reversing polymerization processes to efficiently regenerate monomers from polymers.^1,2^ Polymethacrylates can be depolymerized to monomers at relatively elevated temperatures (400–500 °C) using fluidized bed and high-temperature bath systems.^3−8^ The high temperature fulfills both thermodynamic and kinetic requirements. From a thermodynamic point of view, the system needs to be above the ceiling temperature (*T*_c_) and below the equilibrium monomer concentration ([M]_eq_). As expressed in eqs 1 and 2, the ceiling temperature depends on the monomer concentration [M]_0_, while [M]_eq_ depends on the temperature of the process:
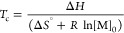
1
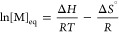
2These equations can be simplified under standard conditions, *T*_c_ = Δ*H*/Δ*S*^0^ at [M]_0_ = 1 mol/L. Values of *T*_c_ are paired with different [M]_eq_ values.^9^*T*_c_ can be reduced under dilute conditions, and the [M]_eq_ can be averted with continuous removal of the monomer from the reaction medium.^10^

In terms of kinetics, the continuous consumption or formation of a monomer requires active chain ends. Therefore, controlled radical polymerization (CRP), also termed reversible deactivation radical polymerization (RDRP), emerges as a powerful tool for efficient depolymerization at moderately lower temperatures because of the preservation of the chain-end functionality (CEF), which can be harnessed to generate active radicals.^9,11^

Among various RDRP methods, Atom Transfer Radical Polymerization (ATRP) and Reversible Addition–Fragmentation chain-Transfer (RAFT) polymerization techniques are most often used to prepare well-defined polymers with low dispersity.^12−20^ While RAFT polymers mainly bear dithiocarbonates, trithiocarbonates, and xanthates at their ω-chain ends, polymers prepared by ATRP contain halogens (i.e., Cl and Br) at the ω-chain ends. Polymethacrylates prepared by ATRP or RAFT have been depolymerized using various activators and/or catalysts at elevated temperatures.^9,11^ Since the polymerization of monomers with bulky substituents is thermodynamically less favorable than those with the smaller ones, their depolymerization can be conducted at lower temperatures. Indeed, the first examples using ATRP were reported for limited/equilibrated polymerization of methacrylates with bulky POSS (polyhedral oligomeric silsesquioxanes) and then for depolymerization of polymethacrylates with large PDMS (poly(dimethylsiloxane)) substituents (up to 79% depolymerization, Scheme 1A).^21,22^

**Scheme 1:**
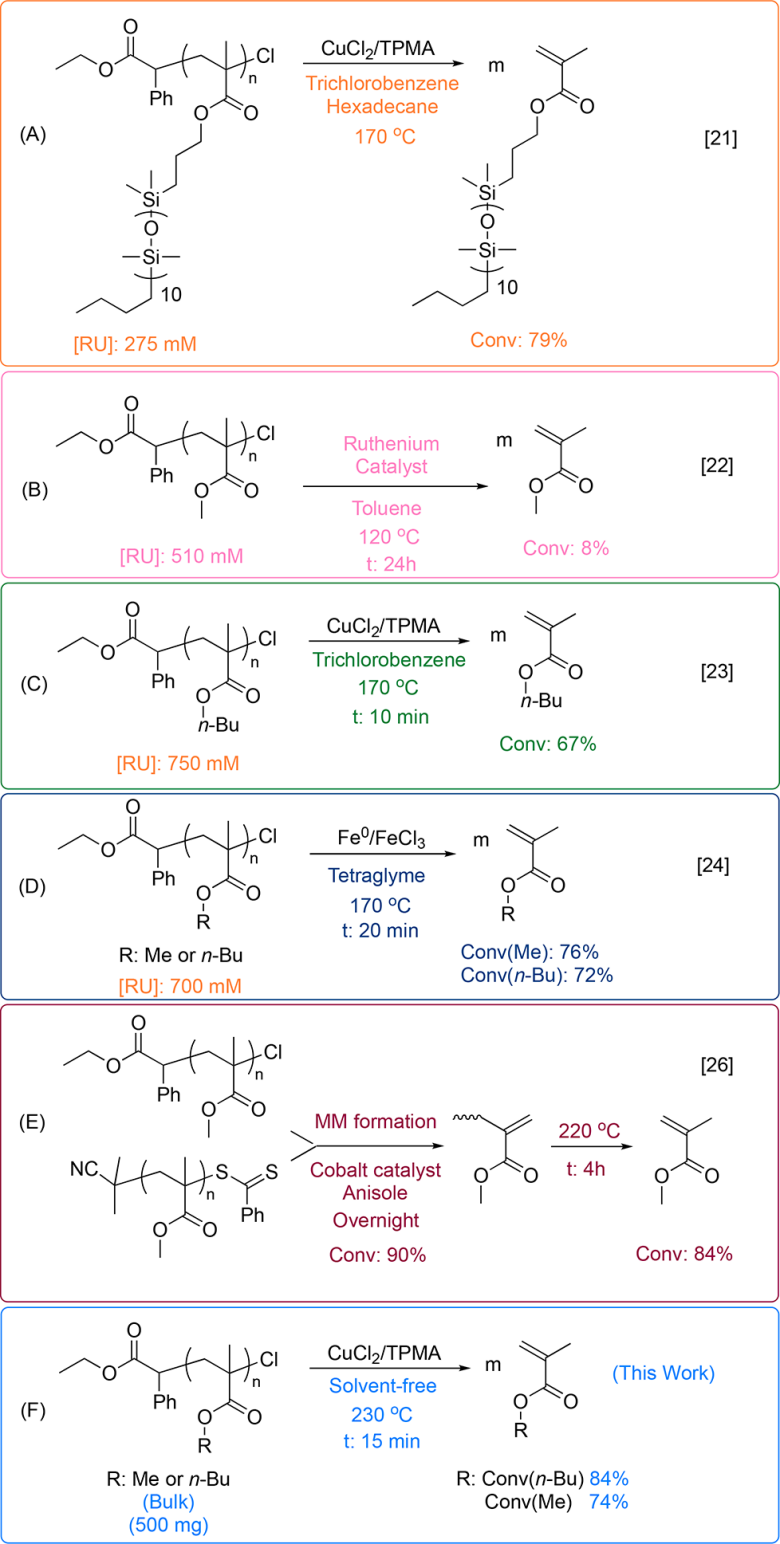
Examples of Depolymerization of Polymethacrylates Prepared by ATRP

The depolymerization of poly(methyl methacrylate) (PMMA) with the smaller methyl group and with terminal chlorine chain end functionality using a ruthenium catalyst yielded only 8% conversion at 120 °C (Scheme 1B).^23^ Depolymerizations of poly(*n*-butyl methacrylate) (PBMA) with terminal chlorine chain-end groups (750 mM repeating unit (RU) concentration) mediated by a copper(II) chloride/tris(2-pyridyl methyl)amine (CuCl_2_/TPMA) catalyst at 170 °C proceeded with 67% yield^24^ (Scheme 1C). The depolymerization of ω-chloro functional PBMA and PMMA ([RU] = 700 mM) mediated by iron chloride salts and iron powder at 170 °C resulted in above 70% depolymerization conversion within 20 min^25^ (Scheme 1D).

Concurrently, the depolymerization of polymethacrylates prepared by RAFT was also studied. Polymethacrylate with PDMS side chains ([RU] = 100 mM) at 70 °C yielded 30% of the monomer.^26^ A higher yield of the monomer up to 92% was achieved at a higher temperature (120 °C), but at a much higher dilution ([RU] = 5.2 mM) and longer times (8 h).^27^ The depolymerizations were strongly accelerated in the presence of light irradiation.^28,29^

The recently reported depolymerization of PMMA with unsaturated chain ends (resembling macromonomers, MM) reached 84% conversion at 220 °C in bulk in 4 h (Scheme 1E). This interesting system requires the conversion of ATRP or RAFT polymers to unsaturated chain ends using a cobalt catalyst and subsequent thermal depolymerization at elevated temperatures. According to TGA analysis, heating for 12 h at 180, 200, and 220 °C, gave 30%, 73%, and 91% depolymerization.^30^ Herein, we report the bulk depolymerization of PMMA and PBMA by the ATRP process. This approach employs chloro-terminated PBMA and PMMA (PBMA-Cl and PMMA-Cl; all experimental information and characterization details about the precursor polymers are provided in the Supporting Information, Figures S1–S12) with CuCl_2_/TPMA catalysts at temperatures between 150 and 230 °C (Scheme 1F). This approach avoids the preparation of macromonomers and is strongly accelerated by a copper catalyst (down to 15 min at 200 and 230 °C). Yields up to 70% TGA and up to 84% on the preparative scale were lower than in ref (30), plausibly due to concurrent lactonization and radical termination.

High-resolution thermogravimetric analysis (HR-TGA) with a dynamic heating rate was first performed under a flow of N_2_. The analysis was performed with a starting heating rate of 5 °C/min, which decreases with weight loss (Figure 1a). HR-TGA of PBMA-Cl showed that the polymer was thermally stable up to temperatures around 350 °C. Small weight loss (2.4%) at 200–250 °C can be attributed to lactonization and the evaporation of BuCl as a byproduct. When 0.022 equiv of CuCl_2_/TPMA (by mole, vs chain end P-Cl) was introduced, a sharp decrease in the mass was observed at 185 °C with a 60% yield of depolymerization. Subsequently, no depolymerization was observed, plausibly due to the loss of chain-end functionality. Nevertheless, the remaining polymer underwent degradation at around 350 °C, similar to the behavior observed in PBMA-Cl without the catalyst. When the concentration of the catalyst was increased to 0.22 equiv vs P-Cl, the depolymerization occurred with a 70% yield at ca. 185 °C, with further complete depolymerization at higher temperatures (350–400 °C). Similar behavior was observed for PMMA-Cl (Figure 1B). The polymer was stable up to 370 °C without the catalyst. However, when 0.022 equiv of the catalyst was present, 60% depolymerization occurred at 185 °C with a 60% yield, followed by further depolymerization above 350 °C.

**Figure 1:**
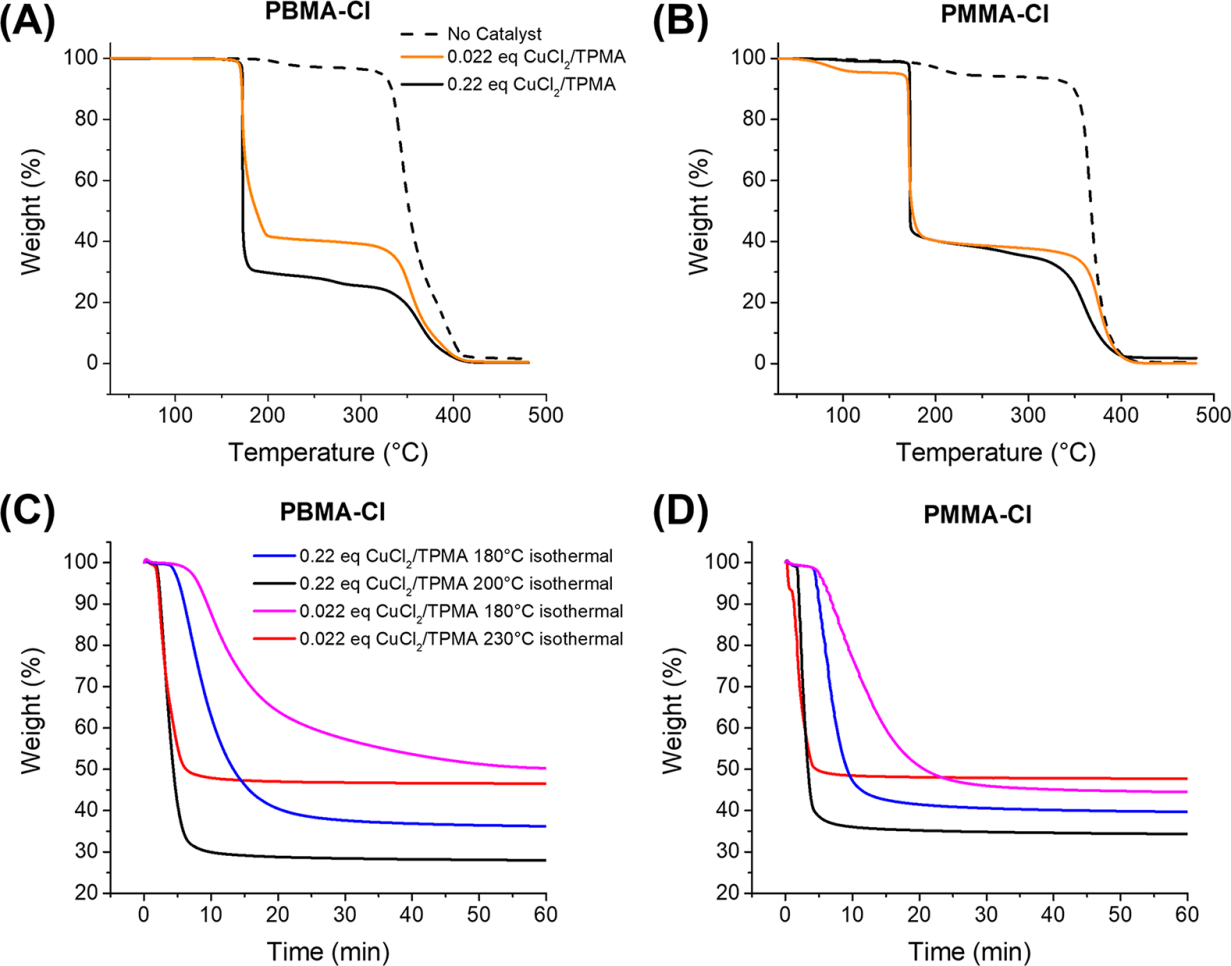
High-resolution TGA analyses with a dynamic heating rate were performed with an initial 5 °C/min heating rate under nitrogen flow. The isothermal analyses were performed by heating the samples at 40 °C/min to the desired temperature. TGA analyses of PBMA-Cl with different catalyst loadings (A); TGA analyses of PMMA-Cl with different catalyst loadings (B); Depolymerization of PBMA-Cl with different catalyst amounts at constant temperatures (C); TGA analyses of PMMA-Cl with different catalyst loadings at constant temperatures (D).

The depolymerization at constant temperatures was then investigated. When PBMA-Cl was heated at 200 °C in the presence of 0.22 equiv CuCl_2_/TPMA, 70% depolymerization was observed within 5 min (Figure 1C). The yield was 62% at 180 °C, with 0.22 equiv catalyst after 20 min. At 180 °C, depolymerization with 0.022 equiv catalysts was significantly slower and yielded ca. 50% after 1 h. A similar yield was observed at 230 °C, but already within 7 min.

In a similar way, PMMA-Cl underwent depolymerization with a yield of 65% at 200 °C with a catalyst loading of 0.22 equiv (Figure 1D). The yield was around 50% already after 5 min when the catalyst amount was reduced to 0.022 equiv, and the temperature was increased to 230 °C. At 180 °C, depolymerization was slower, but yields were higher than at 230 C.

However, incomplete depolymerization indicated a loss of chain end functionality via lactonization, radical termination, and/or catalyst decomposition (*cf. infra*).

It must be noted that the equilibrium monomer concentration for methyl methacrylate is much lower than the observed yields, and it is [MMA]_eq_ = 0.45 M at 180 °C and 2 M at 230 °C, calculated using eq 2 considering Δ*H*_p_ = −56 kJ/mol and Δ*S*_p_ = −117 J/mol °K.^9^

This indicates that the monomer was continuously removed to reach higher yields. This could be due to fast N_2_ flow over the samples in TGA experiments. The faster depolymerization for PMMA could be related to the lower boiling point of MMA (100 °C) compared to BMA (163 °C).

A simple short-path distillation setup was then used to remove monomers formed by depolymerization on a larger preparative scale.^24,25^ Because the glass transition temperatures (*T*_g_) of PBMA and PMMA are below the depolymerization temperatures (150–230 °C), the reactions were carried out in the bulk liquid state, above *T*_g_. A 25 mL round-bottom flask was filled with PBMA-Cl or PMMA-Cl (1 equiv) and CuCl_2_/TPMA (0.22 or 0.022 equiv vs P-Cl) in acetone. Then, acetone was removed using a rotary evaporator prior to depolymerization. The depolymerization was started by placing the flask in an oil bath at different temperatures and applying a vacuum (see Figure S2 for the reaction setup). The pressure during the process was between 2 and 3 mbar. Table 1 shows the yields of the depolymerization processes under different reaction conditions.

**Table 1:** Yields of the Bulk Depolymerization of Polymethacrylates under Different Conditions

run	polymers (P, 0.5 g)	DP^b^	[P-Cl]/[CuCl_2_]/[TPMA]	*T* (°C)	time (min)	conv. (%)^c^	recovered monomer (%)^d^
1	PBMA-Cl	25	1/0.22/0.22	150	60	20	84
2	PBMA-Cl^a^	25	1/0.22/0.22	150	60	35	84
3	PBMA-Br	25	1/0.22/0.22	150	60	8	
4	PBMA-Cl	25	1/0/0	200	60	2.4	
5	PBMA-Cl	25	1/0.22/0.22	200	60	73	92
6	PMMA-Cl	25	1/0.22/0.22	200	60	70	85
7	PBMA-Cl	25	1/0.22/0.22	200	120	74	90
8	PBMA-Cl	60	1/0.22/0.22	200	60	74	82
9	PBMA-Cl^a^	25	1/0.22/0.22	200	60	35	84
10	PBMA-Cl	25	1/0.22/0.22	200	15	74	91
11	PMMA-Cl	25	1/0.22/0.22	200	15	69	85
12	PBMA-Cl	25	1/0.22/0.22	230	15	84	84
13	PMMA-Cl	25	1/0.22/0.22	230	15	73	
14	PBMA-Cl	25	1/0.0022/0.0022	200	60	22	60
15	PBMA-Cl	25	1/0/0.0022/0.0022	230	60	22	62
16	PBMA-Cl^a^	25	1/0.0022/0.0022	200	60	45	82
17	PBMA-Cl	25	1/0.022/0.022	230	60	70	99

a Under UV irradiation (λ = 370 nm).

b Degree of polymerization.

c Calculated based on the loss of polymer weight in the distillation flask.

d Weight fraction of obtained monomer based on the weight loss of the polymer.

First, the depolymerization of PBMA was carried out at 150 °C (runs 1–3). When PBMA-Cl was treated with 0.22 equiv of CuCl_2_/TPMA catalyst, 20% conversion was reached (run 1). Exposure of the reaction mixture to UV irradiation (λ ∼ 370 nm) increased the yield by 15%, and 84% of the monomer was recovered in each case (based on the weight loss of the initial polymer). The increase in depolymerization yield was due to the photoreduction of the Cu(II) complex. When the typical procedure was applied to PBMA-Br, the yield was much lower, 8%, suggesting the more pronounced lactonization with P-Br than with P-Cl end groups.^24^

Then, the temperature was increased to 200 °C. In the absence of a copper catalyst, PBMA-Cl yielded almost no conversion (run 4). However, by introducing 0.22 equiv of CuCl_2_/TPMA, 73% and 70% depolymerization yields were observed in less than 1 h for PBMA-Cl and PMMA-Cl, respectively (runs 5 and 6). When the process was prolonged to 2 h (run 7), the yield did not increase, indicating that the chain ends were dead within an hour, preventing further conversion. With higher molar mass PBMA-Cl (DP = 60), a similar conversion was observed (74%; run 8). A lower conversion was observed when the reaction medium was exposed to UV light at 200 °C (run 9), plausibly due to the excessive generation of radicals. This resulted in more pronounced radical termination and loss of Cl-chain ends, limiting the yield of depolymerization.

In 15 min, the depolymerization was similar to that conducted in 1 h, indicating that the process was essentially completed within 15 min (runs 10 and 11). Reaction at 230 °C yielded 84% depolymerization in <15 min, with 84% monomer recovered (run 12).

Notably, higher depolymerization yields were obtained in the preparative experiments, than by TGA, which can be attributed to the more efficient removal of the monomers from the reaction media, preventing repolymerization and different temperature profiles.

The effect of the catalyst loading was then studied. When PBMA-Cl with a DP = 25 was depolymerized by using 100 times lower copper concentrations, the depolymerization yields decreased (runs 14 and 15). When the same reaction was performed in the presence of UV light (λ = 370 nm), the conversion doubled (45%, run 16). In the case of 10 times less catalyst loading at 230 °C, the yield was comparably higher with an almost quantitative monomer recovery (run 17).

After the preparative depolymerization experiments, the samples were investigated by ^1^H NMR spectroscopy. The collected liquids in the receiving flasks were pure BMA and MMA (<95%; runs 10 and 1, Figures S13 and S14). In the residue, a small amount of BMA and MMA was trapped (∼3.7 and 4.1 mol %, respectively, Figures S15 and S16). Thus, the amount of the residual polymers (24 and 31%) should be further reduced by 3.7% and 4.1% (see Supporting Information for the calculations) to 22 and 27%. This indicates that the overall depolymerization yields in Table 1 are underestimated. Figures S15 and S16 also provided information on the structures of the residual polymers. The disproportionation yields were calculated by comparing the integrated areas of chain-end vinyl protons to those of main chains. The remaining chain ends were assigned to the lactonization product. Accordingly, the % contributions of the processes are as follows: for PBMA depolymerization = 77.7%, disproportionation = 9.6%, and lactonization = 12.7%. For PMMA, the % contributions of the processes are depolymerization = 73%, disproportionation = 7.4%, and lactonization = 19.6% (see Supporting Information). By comparison to PMMA, lactonization in PBMA is diminished for the larger butyl group in comparison with the smaller methyl group.

The high temperatures could also lead to the elimination of HBr and the generation of unsaturated chain ends, i.e., macromonomers (cf. ref (30)). However, the depolymerization catalyzed by Cu complexes was much faster, indicating ATRP-type activation–deactivation and depolymerization mechanism. Experiments with CuCl/TPMA and Cu(0) resulted in a very low depolymerization yield, possibly due to the formation of too high radical concentrations that enhanced radical termination reactions. In separate experiments, depolymerizations were studied using PBMA-Cl with CuCl_2_ (without TPMA ligand) and PBMA prepared by free-radical polymerization (no R-Cl chain end), subsequently adding CuCl_2_/TPMA. Each displayed less than 3% weight loss at 200 °C. This suggests that both the chain-end functionality (R-Cl) and CuCl_2_/TPMA complex are essential for depolymerization.

The Cu catalyst stability was studied by UV at elevated temperatures after heating to 135 and 175 °C for 15 min in propylene carbonate. Obvious changes in the color of CuCl_2_/TPMA solutions were visually observed (Figure 2A). The decomposition of the complex starts already at 135 °C, as evidenced by the decrease in the absorption at ∼930 nm. The reduction was even faster at 175 °C. When the UV cell was purged with air, some of the plausibly formed Cu(I) was reoxidized (Figure 2B). The decomposition of the ligand may lead to the formation of bare CuCl_2_, with a smaller absorption extinction coefficient.

**Figure 2:**
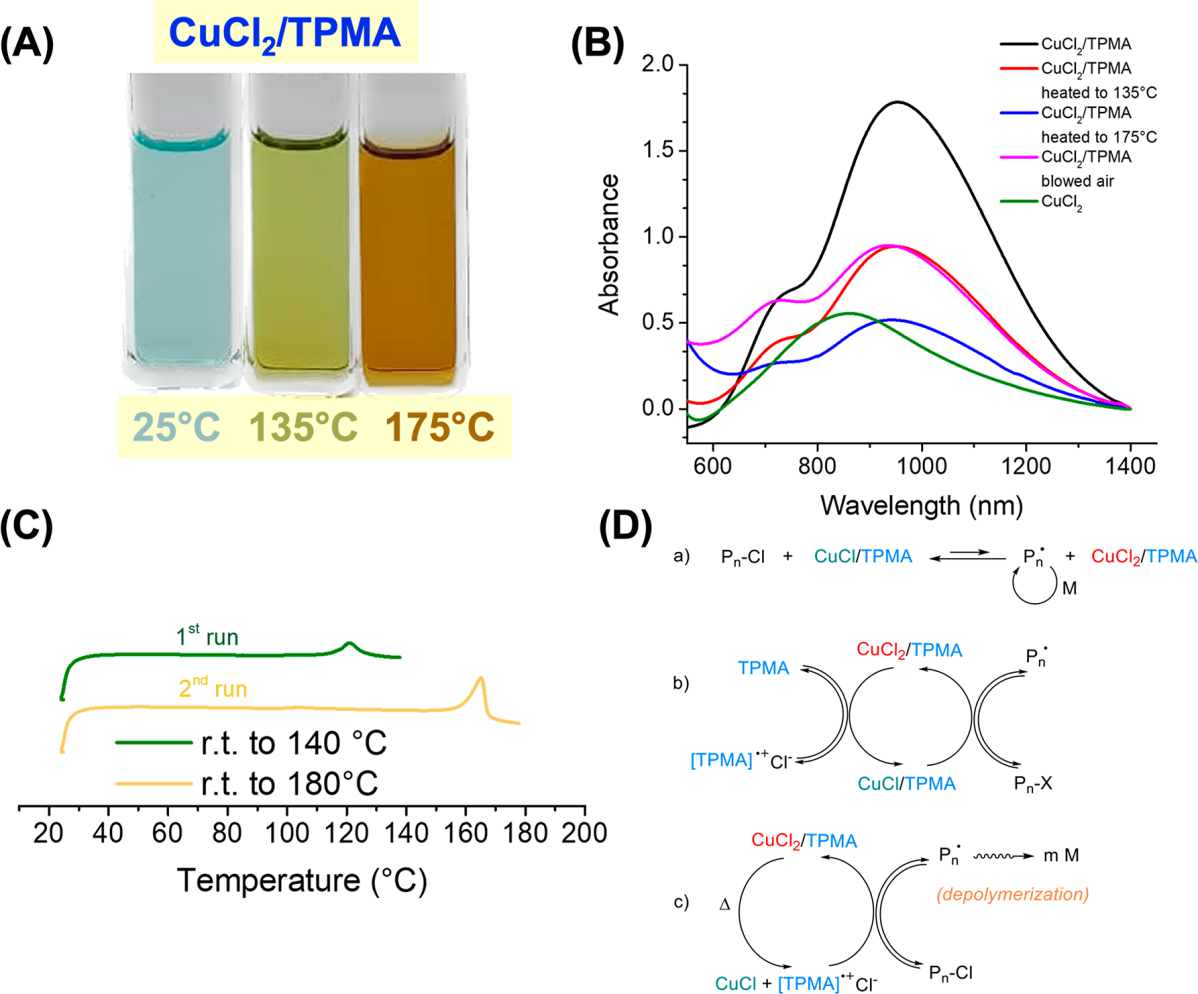
(A) Change of color of a solution of CuCl_2_/TPMA upon heating at 135 and 175 °C in propylene carbonate. (B) UV–vis spectra of CuCl_2_ and CuCl_2_/TPMA (0.016 M) under different conditions. (C) DSC thermogram of CuCl_2_/TPMA complex. (D) Classical ATRP mechanism using CuCl/TPMA (a), ARGET ATRP (b), and the postulated depolymerization mechanism (c).

The thermal behavior of the CuCl_2_/TPMA complex was studied by differential scanning calorimetry (DSC; Figure 2C). An endotherm was observed when the complex was heated to around 135 °C, which might be attributed to melting, as visually confirmed by an experiment conducted in a melting point apparatus. In the second cycle of heating, another endotherm at around 170 °C was observed, which can be related to the decomposition of the complex.

It should be stressed that the depolymerization was studied using the CuCl_2_/TPMA complex, which is a typical radical deactivator at ambient temperatures, and it should not activate dormant alkyl chlorides to generate radicals needed for depolymerization (Figure 2D(a)). However, it was reported that the depolymerization of Cl-terminated polymethacrylates at elevated temperatures also slowly occurred in the presence of a free ligand TPMA.^24^ This could be due to the outer sphere electron transfer and formation of a radical and halide anion in a concerted process or a radical anion at the chain end (dissociating to halide anion and a radical capable of depolymerization) along with the TPMA radical cation. Alternatively, halogen bonding between alkyl halides and polydentate amine ligands may facilitate the formation of radicals.^31^ Therefore, the process could follow a mechanism similar to ARGET, with TPMA as reducing agent or SARA^32^ with additional supplemental activation by TPMA (Figure 2D(b)).

Under typical depolymerization conditions, four possible processes should be considered: (i) depolymerization/polymerization, (ii) termination, (iii) ATRP activation–deactivation equilibria, and (iv) lactonization. While the first three require the generation of radicals at the chain end, the latter can proceed without an external activator (Scheme S1). They are supplemented by the evolution of the complex stability and by the removal of the monomer from the reaction mixture. Kinetic parameters, activation energies, and mass transport phenomena of these processes should be determined. A delicate interplay between these reactions should be further studied to maximize the depolymerization efficiency.

In summary, a fast bulk ATRP depolymerization catalyzed by CuCl_2_/TPMA complexes of Cl-terminated PBMA and PMMA is reported. Yields of depolymerization were up to 84% with almost quantitative recovery of the available monomer within 15 min at temperatures between 180 and 230 °C. The reactions were tested analytically and then carried out at the gram scales at temperatures above glass transition temperatures.

## Supporting Information Available

Details of experimentation and instrumentation and spectral and chromatographic analyses (PDF)

## Dedication

This work is dedicated to the memory of Professor Yusuf Yagci who has recently passed away.
