# Supplementary material for: Fast Bulk Depolymerization of Polymethacrylates by ATRP
Source: ACS Macro Lett. 2023 Aug 2;12(8):1173–8. doi: 10.1021/acsmacrolett.3c00389 (PMC10433507; doi:10.1021/acsmacrolett.3c00389)
Supplement: Supplementary file 1 — mz3c00389_si_001.pdf [file mz3c00389_si_001.pdf]

Supporting Information

Fast Bulk Depolymerization of Polymethacrylates by ATRP

Ferdinando De Luca Bossa, Gorkem Yilmaz, Krzysztof Matyjaszewski*

Department of Chemistry, Carnegie Mellon University, 4400 Fifth Avenue, Pittsburgh, Pennsylvania 15213, United States

Corresponding Author: km3b@andrew.cmu.edu

Experimental Part

Materials

Methyl methacrylate (MMA, Sigma-Aldrich, 99%) and *n*-butyl methacrylate (BMA, Sigma-Aldrich, 99%) were purified by passing through basic alumina. Copper (II) chloride ($CuCl_2$, 98%, Sigma-Aldrich) was dried in a Schlenk flask under vacuum at 165 °C for 18 h to remove hydrated impurities. Ethyl α-chlorophenyl acetate (ECPA, Sigma-Aldrich, 97%) was used as received.

Instrumentation

The apparent molecular weights (M_n) and dispersity (*Đ*) were measured relative to poly(methyl methacrylate) (PMMA) standards by gel permeation chromatography (GPC) conducted with a Waters 515 pump and Waters 2414 differential refractometer using PSS columns (SDV 10^5, 10^3, 500 Å) with THF as eluent at 35 °C and at a flow rate of 1 mL·min^{-1}. ^{1}H NMR spectra were collected using a Bruker Advance 500 MHz NMR spectrometer with $CDCl_3$ as a solvent at room temperature. The Differential Scanning Calorimetry analysis was performed with a Perkin Elmer DSC 4000 using a rate of heating 10 °C/min. Thermogravimetric Analysis (TGA) was performed with a TA TGA 550 using high-resolution mode.

Deconvolutions

The deconvolution of macroinitiators chain extensions GPC traces was performed with ORIGIN software. The GPC trace was converted from weight distribution to number distribution in order to highlight the presence of small peaks corresponding to dead initial polymers.

Synthetic Procedures

Synthesis of PBMA-Cl

The starting molar ratios for the synthesis of PBMA were [BMA]/[ECPA]/[$CuCl_2$]/[PMDETA]/[$Sn(EH)_2$] = 60/1/0.1/0.175/0.05.

BMA (39.2 g, 0.275 mol), ECPA (912 mg, 459 mmol), $CuCl_2$ (61.7 mg, 0.459 mmol), PMDETA (139 mg, 0.803 mmol), tin(II) 2-ethylhexanoate (93 mg, 0.230 mmol, was added as 1 ml of a stock solution 0.093 g/ml in DMF), and MeCN (17 ml) was added in a 100 ml Schlenk flask. The resulting solution was degassed by three cycles of freeze-pump-thaw. The flask was backfilled with nitrogen at the last cycle and, after thawing the solution, the flask was put in an oil bath at 70°C for 17.5h. After 17.5 h, the conversion calculated by NMR was 81%. Multiple precipitations in a mixture of ice/methanol isolated the polymer. The calculated DP from the ^{1}H NMR of the pure product was 61 ($M_{n,GPC}$ = 9400, $Đ$: 1.14, Figure S1-2).

Synthesis of PBMA-Cl (DP=25)

PBMA with shorter length was synthesized using the same procedure but stopping the reaction earlier at 40% conversion to obtain a polymer with DP=26, M_n= 3900, Đ=1.17 (Figure S5-6)

Synthesis of PMMA-Cl

PMMA was synthesized using the same procedure but with slightly different initial molar ratios ([MMA]/[ECPA]/[$CuCl_2$]/[PMDETA]/[$Sn(EH)_2$ = 50/1/0.1/0.175/0.05). The DP of PMMA-Cl calculated from the NMR spectra of the pure polymer recorded in $CDCl_3$ was 25 ($M_{n,GPC}$ = 2700, $Đ$: 1.14, Figure S9-10).

Chain Extension Experiments

The chain extension reactions were performed with both PBMA-Cl and PMMA-Cl using identical polymerization conditions except using the obtained polymers as initiators. The targeted DPs were 25 times those of the macroinitiators. The GPC analyses demonstrated that the chain-end fidelity of the precursor polymers was almost quantitative, as no initial polymers were observed after chain extensions.

(Figure S7,S8, S11, S12) Only in the case of PBMA DP= 61 the chain initiation efficiency was around 90%. (Figure S3 and 4).

Bulk Depolymerization

In a 25 ml round bottom flask was added the macroinitiator, the catalyst, and the amine in acetone. Acetone was removed using a rotary evaporator. Then, the flask was weighed prior to depolymerization. The depolymerization was started by placing the flask in an oil bath at controlled temperatures under a vacuum. The receiving flask was placed in liquid nitrogen in the short distillation path to collect the generated monomers.

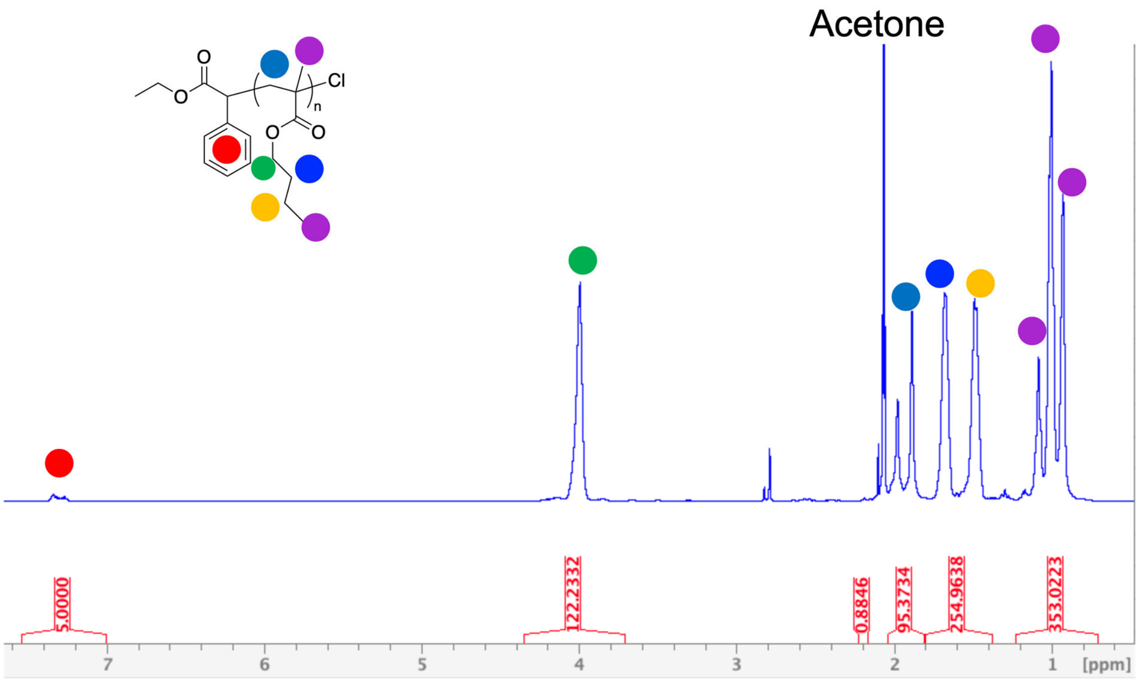

Figure S1. ^{1}H NMR of PBMA-Cl in d_6-Acetone. DP calculated 61 (Run 8).

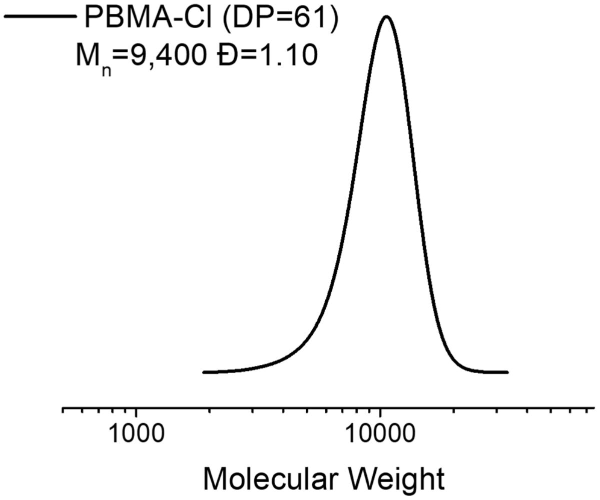

Figure S2. GPC traces of PBMA-Cl (DP=61) recorded in THF (Run 8).

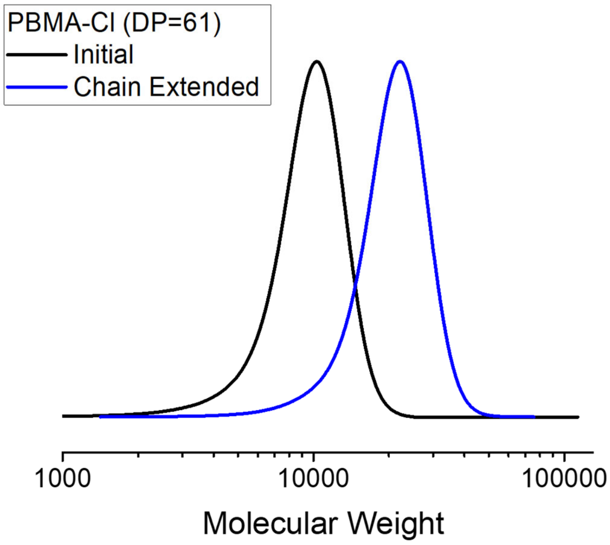

Figure S3. Chain extension of PBMA-Cl (DP=61) recorded in THF (Run 8).

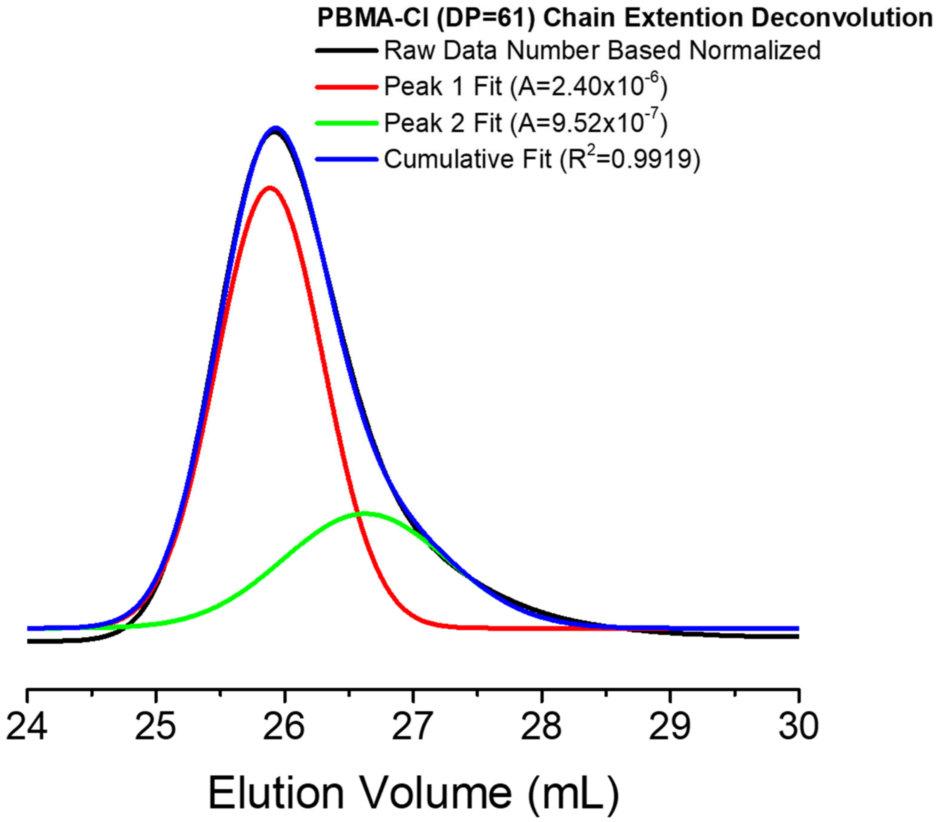

Figure S4. Chain extension deconvolution of PBMA-Cl (DP=61). Chain initiation efficiency (CIE) = 72%. (If the error in the deconvolution is considered, it is possible to reach up to 84% CIE which is in agreement with what we obtained from the depolymerization in the main text, Run 8, 74% conversion)

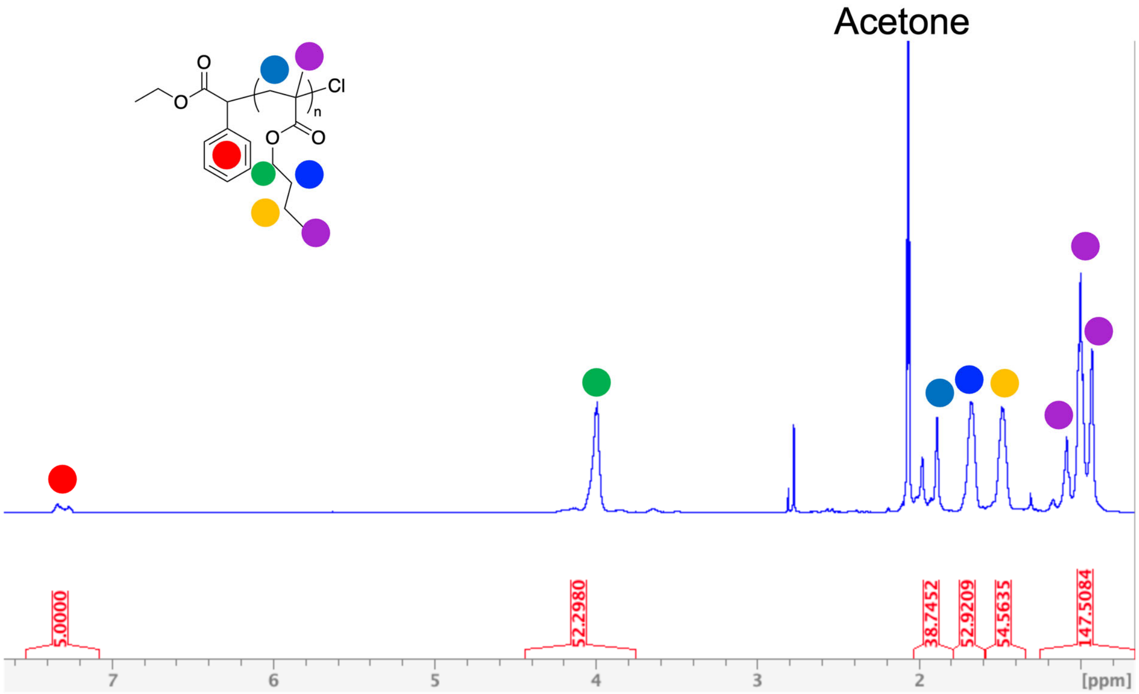

Figure S5. ^{1}H NMR of PBMA-Cl in d_6-Acetone. DP calculated 26 (Runs 1, 2, 4, 5, 7, 9, 10, 12,14-16).

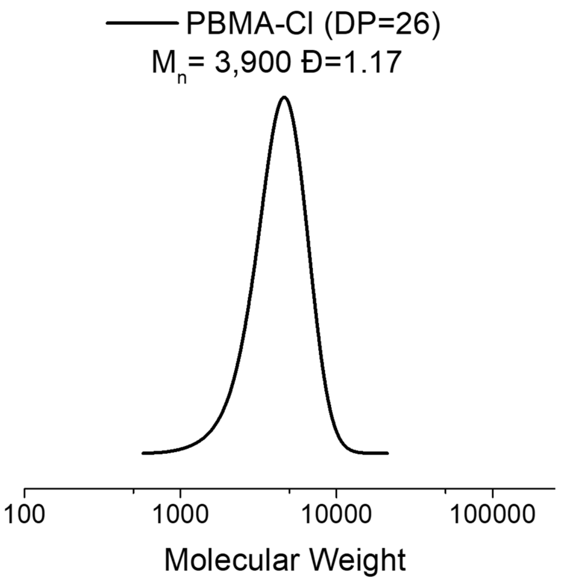

Figure S6. GPC traces of PBMA-Cl recorded in THF (Runs 1, 2, 4, 5, 7, 9, 10, 12, 14-16).

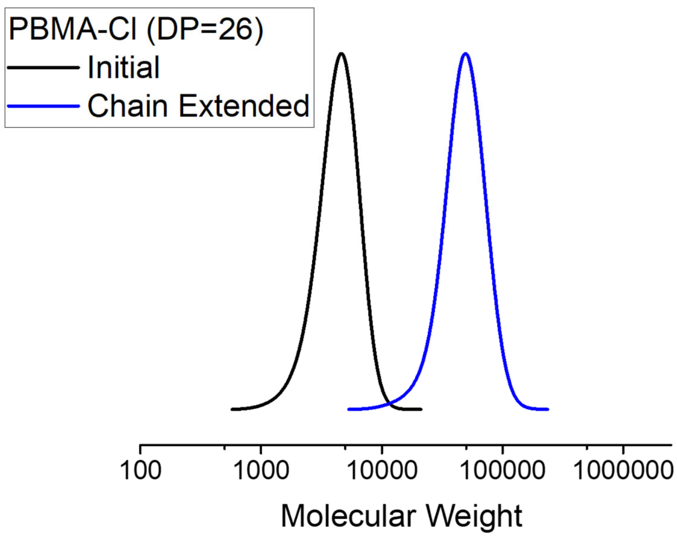

Figure S7. Chain extension of PBMA-Cl (DP=26) recorded in THF (Runs 1, 2, 4, 5, 7, 9, 10, 12, 14-16).

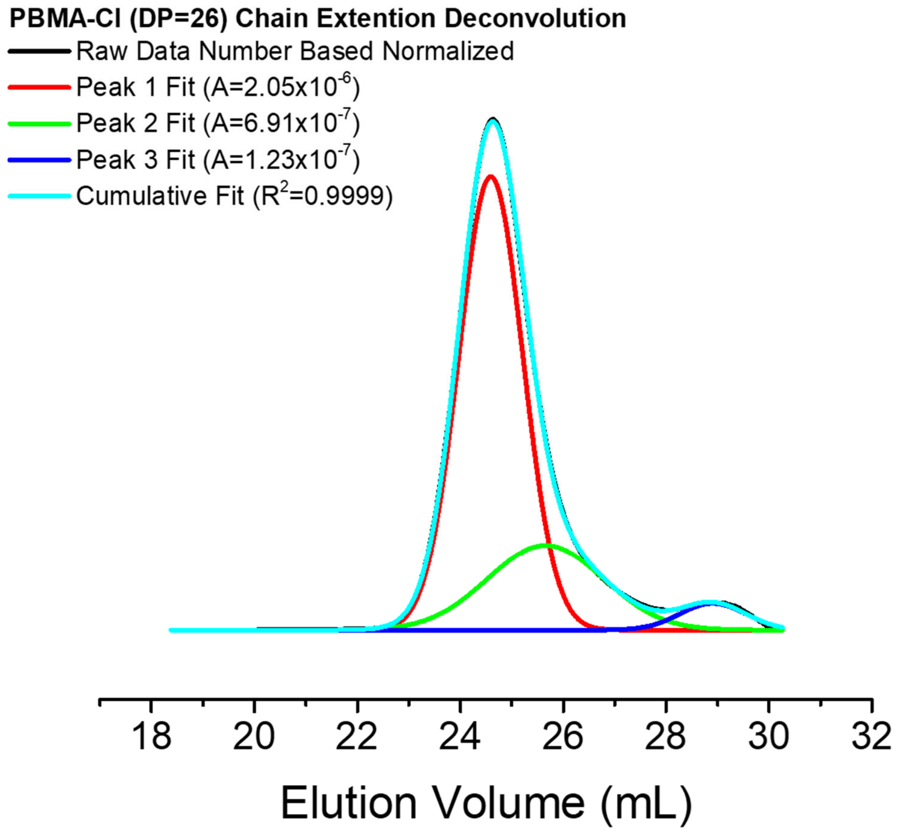

Figure S8. Chain extension deconvolution of PBMA-Cl (DP=26). Chain initiation efficiency (CIE) = 96% (Runs 1,2,4,5,7,9,10,12,14-16). The deconvolution was performed considering three Gaussian peaks to replicate the tailing of the curve between 26-28 ml elution volume. The only peak related to not initiated polymer is peak 3 (blue)

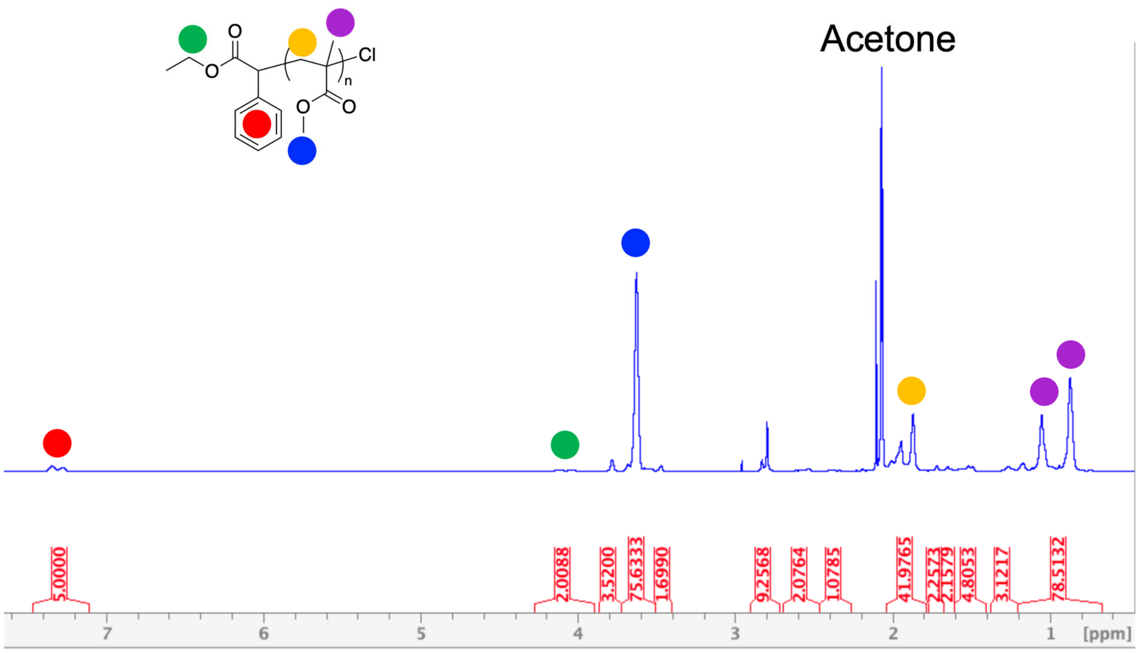

Figure S9. ^{1}H NMR of PMMA-Cl in d_6-Acetone. DP calculated 25 (Run 6, 11, 13).

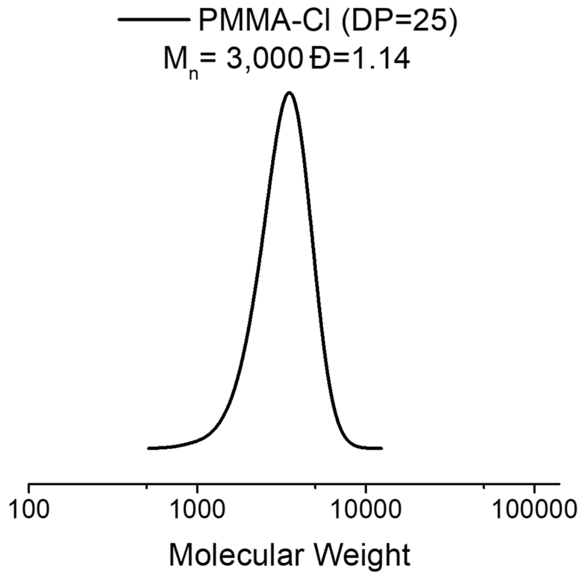

Figure S10. PMMA-Cl GPC trace recorded in THF (Run 6, 11, 13).

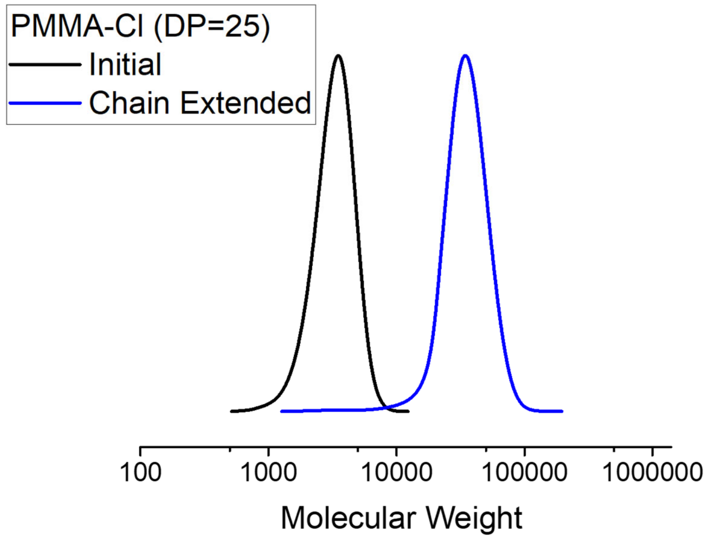

Figure S11. Chain extension of PMMA-Cl (DP=25) recorded in THF (Run 6, 11, 13).

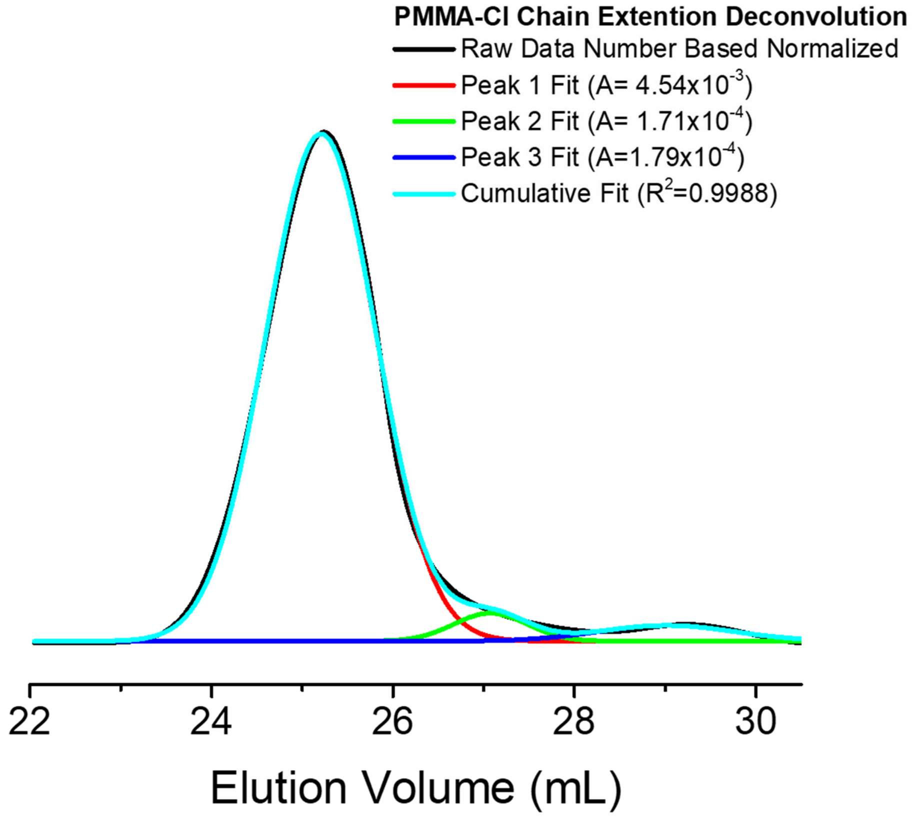

Figure S12. Chain extension deconvolution of PMMA-Cl (DP=25). CIE=96% (Runs 6, 11, 13). The deconvolution was performed considering three Gaussian peaks to replicate the tailing of the curve between 27-28 ml elution volume. The only peak related to not initiated polymer is peak 3 (Blue)

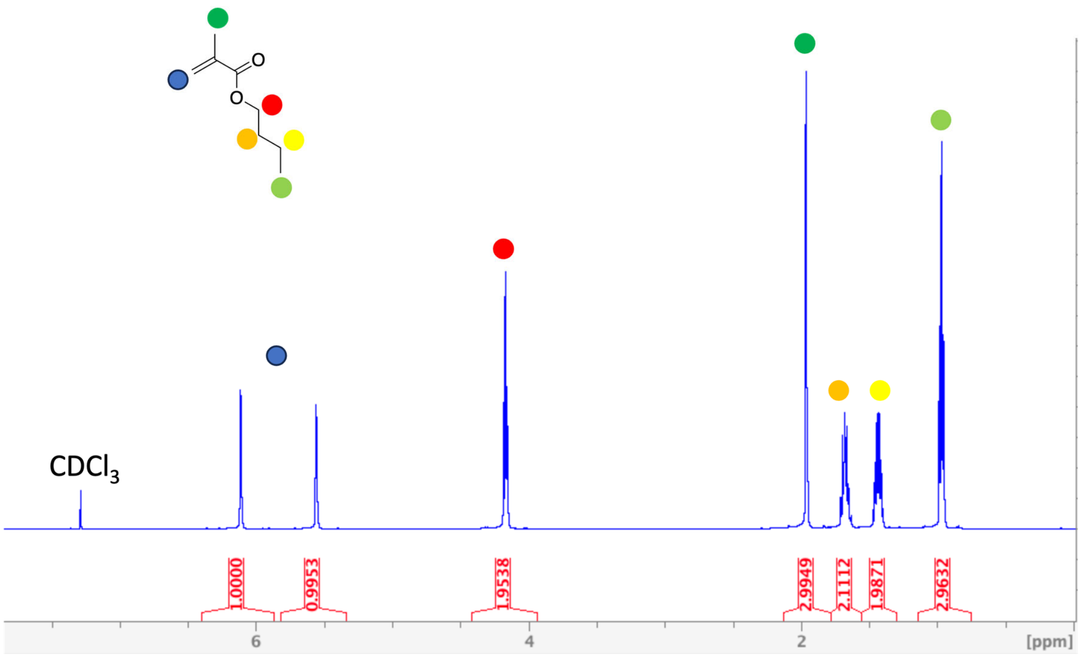

Figure S13. ^{1}H NMR of depolymerized BMA in $CDCl_3$ without purification (Run 10 main text). The product (BMA) appears pure by ^{1}H NMR without impurities.

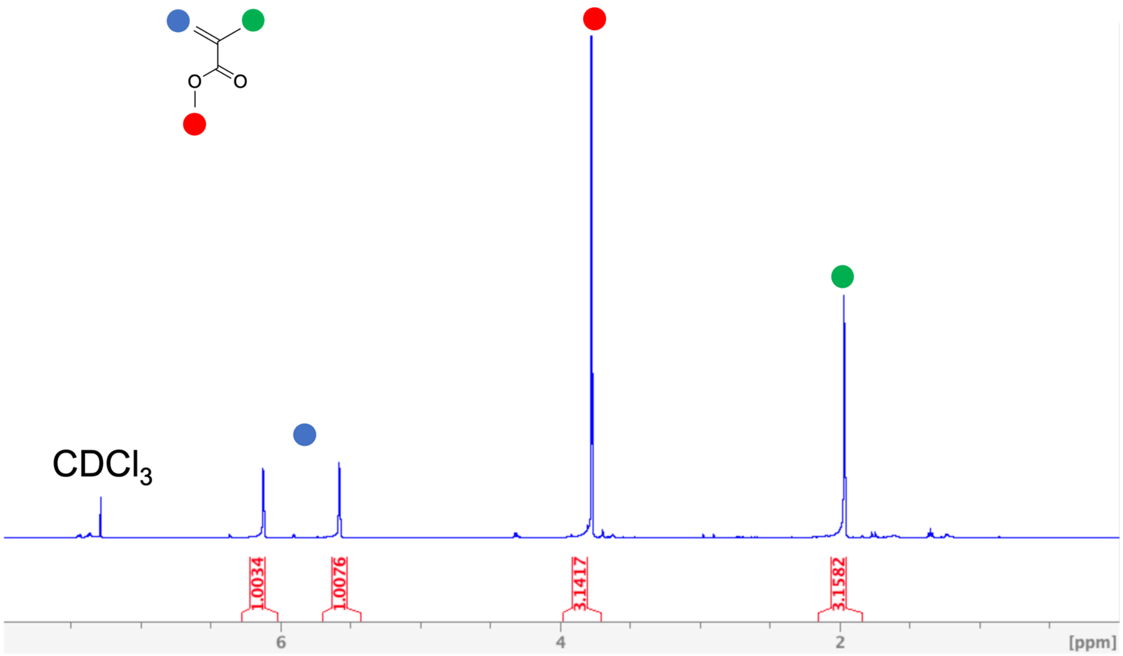

Figure S14. ^{1}H NMR of depolymerized MMA in $CDCl_3$ without purification (Run 11 main text).

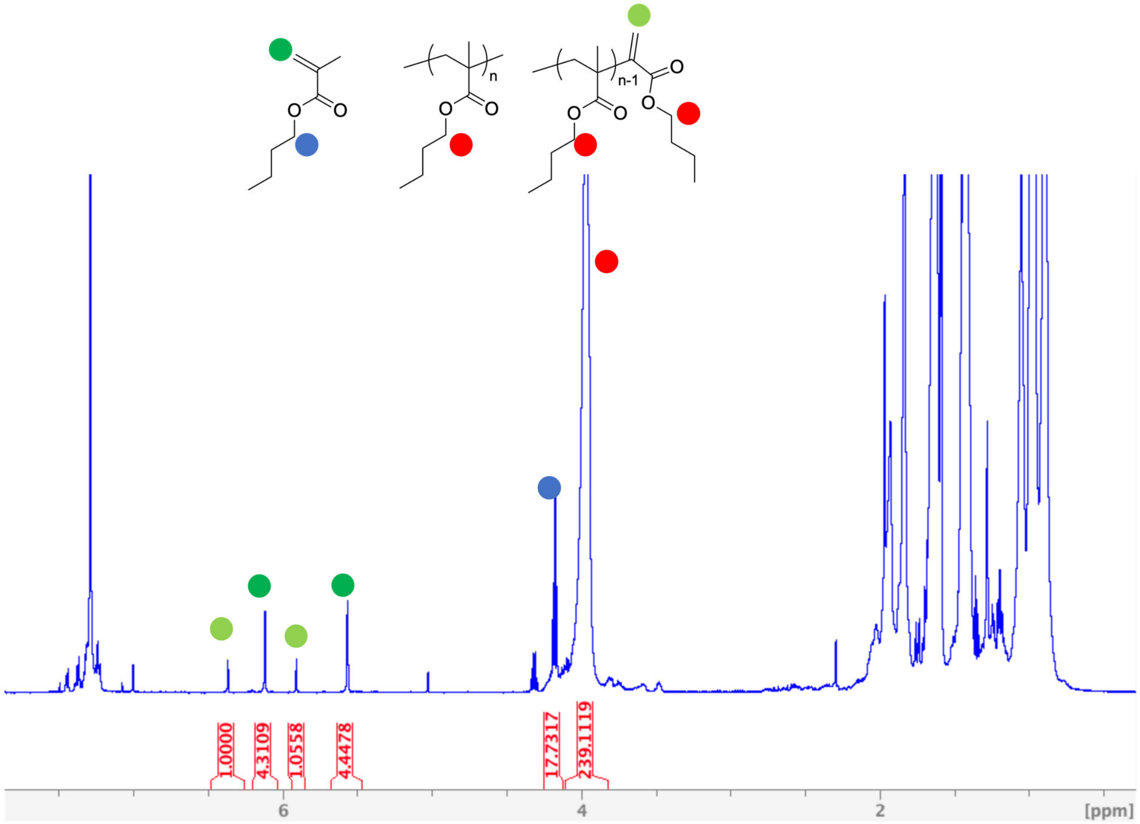

Figure S15. ^{1}H NMR of PBMA after depolymerization (Run 10 main text) in $CDCl_3$ without purification. 3.7% of the monomer was trapped in the polymer residue.

Calculation of contributions of depolymerization, termination by disproportionation and lactonization for PBMA

The ^{1}H NMR analysis of the residual PBMA provides information on the contributions of the involved depolymerization and termination processes. Termination in radical polymerization of methacrylates is dominated by disproportionation over recombination. The level of disproportionation increases with temperature and at 200°C exceeds 96%.[1,2] Therefore, we neglected the contribution of termination by recombination in the analysis below.

The residual PBMA had DP=25, meaning that the total amount of terminal groups (P^T) should be 4%, which should include lactone, vinyl and hydrogen end groups: P^L, $P^=$, P^H.

Integrations of vinyl end groups ([(1 + 1.06) / 2] = 1.03). vs pendant α-methylene groups (239.1 / 2) indicate 0.86 % of unsaturated chain ends.

The disproportionation should yield the same amount of unsaturated and saturated chain ends, so $P^= = P^H = 0.86\%$

This indicates $P^L = P^T – (2 \times P^=) = 2.28\%$

By normalizing P^T to 100%, values of $P^= = P^H = 21.5\%$ and $P^L = 57\%$ were calculated for the residual polymer.

The percentage of the remaining monomer was calculated by comparing the integrated ratios of the vinyl hydrogen peaks of the monomer with those of pendant **α**-methylene protons.
The average integrated ratio of vinyl protons of the monomer {(4.31 + 4.45)/2 = 4.38} over the integral area of pendant **α**-methylene protons (239.1/2):
(4.38/119.55) × 100% = 3.7%
The overall depolymerization yield measured as a weight loss (74%) should be adjusted by adding the remaining/trapped monomer (3.7%) to calculate the amount of the actual residual polymer = 100% - (74% + 3.7%) = 22.3%
Therefore, this enables the calculation of the contribution of the processes involved:
Depolymerization: 74% + 3.7% = 77.7%
Termination by disproportionation: 22.3% x (21.5% + 21.5%) = 9.6 %
Lactonization: 22.3% x 57% = 12.7%

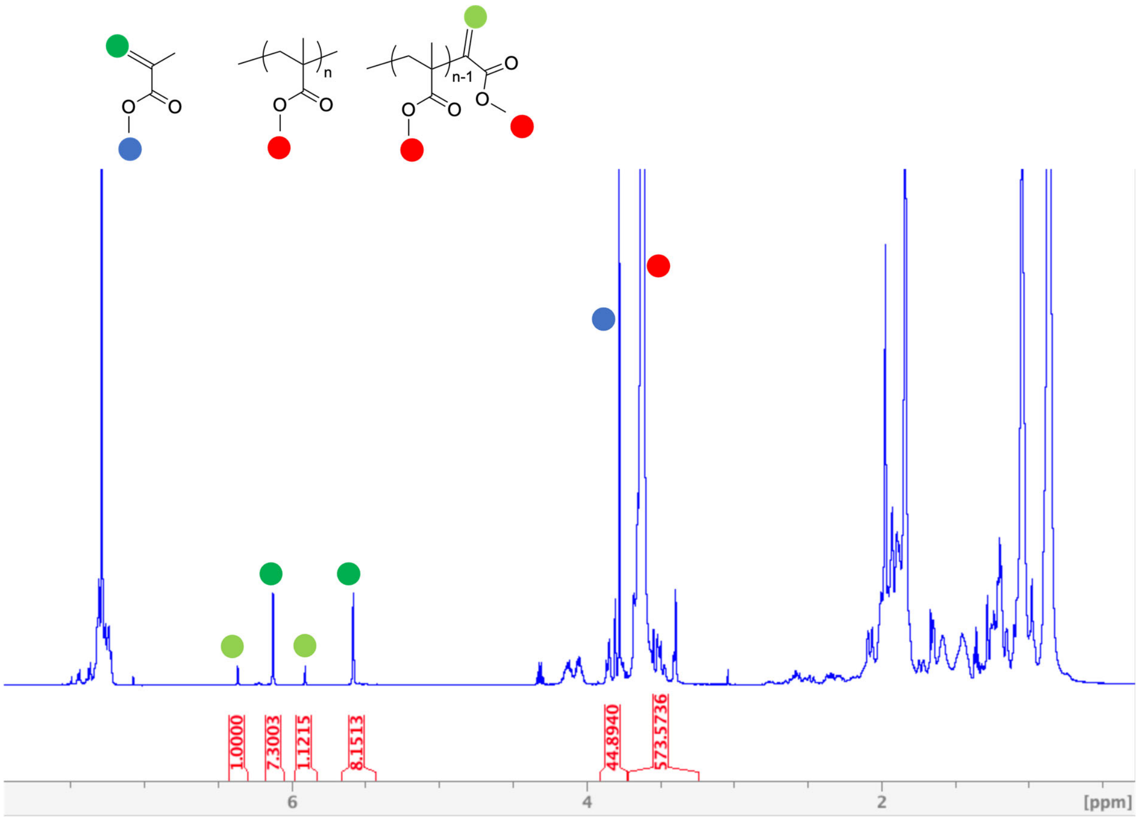

Figure S16. ^{1}H NMR of PMMA after depolymerization (Run 11 main text) in $CDCl_3$ without purification. 4.1% of the monomer was trapped in the residual polymer.

Calculation of contributions of depolymerization, termination by disproportionation and lactonization for PMMA

The ^{1}H NMR analysis of the residual PMMA provides information on the contributions of the involved depolymerization and termination processes. Termination in radical polymerization of methacrylates is dominated by disproportionation over recombination. The level of disproportionation increases with temperature and at

200°C exceeds 93.5%.[3-5] Therefore, we neglected the contribution of termination by recombination in the analysis below.

The residual PMMA had DP=25, meaning that the total concentration of terminal groups (P^t) is 4%, which are lactone, vinyl or hydrogen end groups: P^L, $P^=$, P^H.

Integration of vinyl end groups ((1.00 + 1.12)/2 = 1.06) vs methoxy groups (573.6/3) indicates 0.55 % of unsaturated chain end.

The disproportionation yields the same amount of unsaturated and saturated chain ends $P^= = P^H = 0.55\%$

This indicates $P^L = P^t - (2 \times P^=) = 2.9\%$

By normalizing P^T to 100%, one can obtain values of $P^= = P^H = 13.75\%$ and $P^L = 72.5\%$

The percentage of the remaining monomer was calculated by comparing the integrated ratios of one of the vinyl hydrogen peaks of the monomer with those of pendant methoxy protons.

The average integrated ratio of vinyl protons of the monomer {(7.3 + 8.2) / 2 = 7.8} over the integral area of pendant methoxy protons (573.6/3=191.2):

(7.8 / 191.2) × 100% = 4.1 %

When the depolymerization conversion is considered by adding the remaining monomer (4.1%) to the depolymerization yield (69%), the disproportionation level can be calculated as follows:

residual polymer = 100% - (69% + 4%) = 27%

Therefore, this enables the calculation of the contribution of the processes involved:

Depolymerization: 69% + 4% = 73%

Termination by disproportionation: 27% x (13.75% + 13.75%) = 7.4 %

Lactonization: 27% x 72.5% = 19.6%

Scheme S1. Competing termination reactions for polymethacrylate-Cl depolymerization.

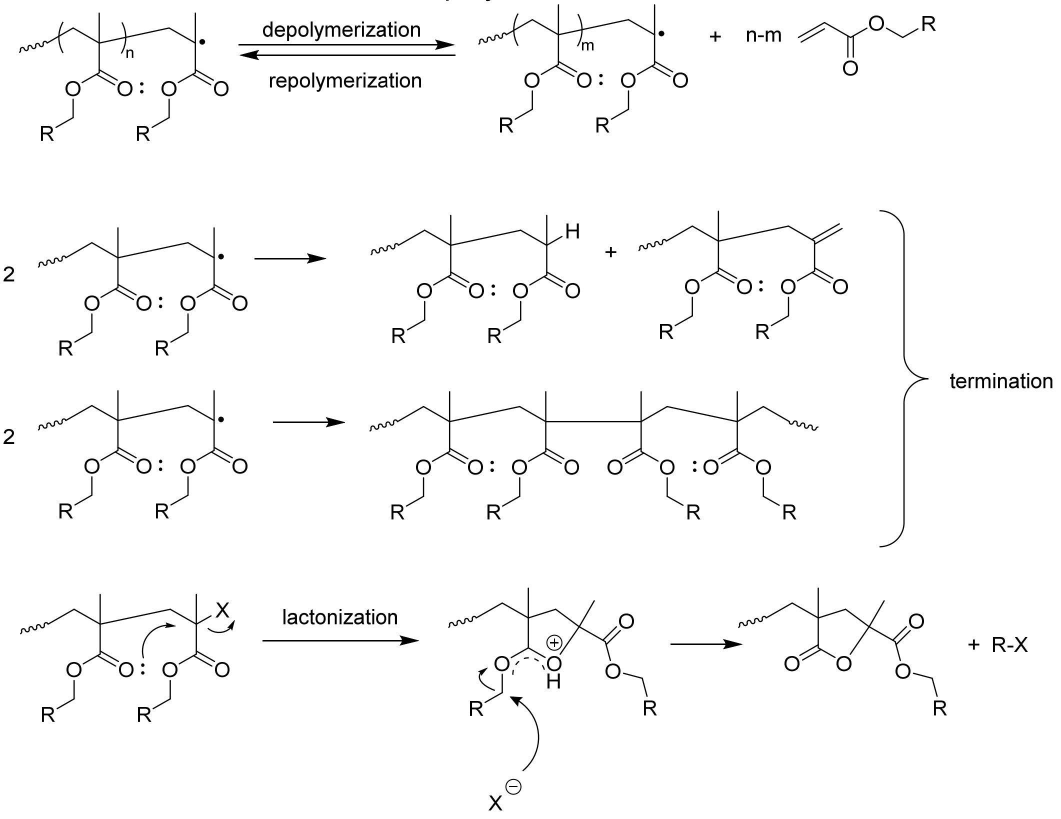

References

1. C. H. Bamford, R. W. Dyson, G. C. Eastmond, *Polymer* 1969, **10**, 885.
2. G. Ayrey, A. C. Haynes, *Eur. Polym. J.* 1973, **9**, 1029.
3. J. C. Bevington, H. W. Melville, and R. P. Taylor, J. Polym. Sci. 14, 463 (1954)
4. C. H. Bamford, G. C. Eastmond, and D. Whittle, Polymer 10, 771 (1969)
5. M. D. Zammit, T. P. Davis, D. M. Haddleton, and K. G. Suddaby, Macromolecules 30, 1915 (1997)
